# Messengers From the Gut: Gut Microbiota-Derived Metabolites on Host Regulation

**DOI:** 10.3389/fmicb.2022.863407

**Published:** 2022-04-22

**Authors:** Chenyu Li, Yaquan Liang, Yuan Qiao

**Affiliations:** Division of Chemistry and Biological Chemistry, School of Physical and Mathematical Sciences, Nanyang Technological University, Singapore, Singapore

**Keywords:** gut microbiota-derived metabolites, short-chain fatty acids, bile acids, peptidoglycan fragments, adaptive and innate immunity, host homeostasis

## Abstract

The human gut is the natural habitat for trillions of microorganisms, known as the gut microbiota, which play indispensable roles in maintaining host health. Defining the underlying mechanistic basis of the gut microbiota-host interactions has important implications for treating microbiota-associated diseases. At the fundamental level, the gut microbiota encodes a myriad of microbial enzymes that can modify various dietary precursors and host metabolites and synthesize, *de novo*, unique microbiota-derived metabolites that traverse from the host gut into the blood circulation. These gut microbiota-derived metabolites serve as key effector molecules to elicit host responses. In this review, we summarize recent studies in the understanding of the major classes of gut microbiota-derived metabolites, including short-chain fatty acids (SCFAs), bile acids (BAs) and peptidoglycan fragments (PGNs) on their regulatory effects on host functions. Elucidation of the structures and biological activities of such gut microbiota-derived metabolites in the host represents an exciting and critical area of research.

## Introduction

Over millions of years of evolution, humans have developed a mutualistic relationship with approximately 3.8 × 10^13^ individual microbes from nearly 1,000 different species in the human intestinal tract, collectively known as the gut microbiota (Ley et al., 2006; Qin et al., 2010; Almeida et al., 2019). At the fundamental level, the human host provides habitats and nutrients to the resident gut microbes, while the microbiota aids in the breakdown of complex carbohydrate fibers that are otherwise indigestible by the host and synthesizes essential vitamins, co-factors, and metabolites for the human body. Thus, the gut microbiota mediates the interactions between indigested food and the host body. The host’s dietary choices and lifestyles can significantly impact the gut microbiota, shaping both the microbial community and the pools of microbiota-derived metabolites (David et al., 2014; Singh et al., 2017; De Angelis et al., 2019; Zmora et al., 2019). While a balanced gut microbiota is critical for maintaining normal host functioning, perturbations to the gut microbiota and their metabolites, known as gut dysbiosis, can have deleterious effects on the host, inflicting a wide range of diseases including inflammatory, metabolic, and neurodegenerative diseases (Cox et al., 2015; Ni et al., 2017; Weiss and Hennet, 2017; Martin et al., 2018).

Remarkably, more than 10% of metabolites in host systemic circulation are of the gut microbiota origin, which act as ligands or hormones that interact with specific host receptors both locally and systemically to modulate downstream effects in the host (Wikoff et al., 2009). In recent years, an increasing number of studies have used untargeted metabolomics to discover novel metabolites of the gut microbiota and focused on deciphering the biological functions of specific metabolites produced by individual gut bacterial strains. In this review, we discuss current knowledge on the roles of gut microbiota-derived metabolites on host health, with a particular focus on three prominent families: short-chain fatty acids (SCFAs), bile acids (BAs), and peptidoglycan fragments (PGNs). Understanding the impact of gut microbiota-derived metabolites on host health is critical for developing potential therapeutic strategies against microbiota-associated diseases.

## Short-Chain Fatty Acid

The symbiotic microbes residing in the host gut lumen play the essential function of breaking down dietary carbohydrate fibers indigestible by the host, producing a large amount of short-chain fatty acids, including acetate, propionate, and butyrate as the metabolic end-products. The concentration of SCFAs is estimated to reach 50–100 mM in the colon (Cummings et al., 1987). These small-molecule metabolites are carried by host systemic circulation to reach extraintestinal organs and make broad-range impacts on the host (Silva et al., 2020; Gautier et al., 2021). In particular, SCFAs provide the energy source for colonocytes, mediate host homeostatic response, and participate in gut-brain crosstalk (Vijay and Morris, 2014; Schönfeld and Wojtczak, 2016). As SCFAs are commensal fermentation products, their levels and compositions in the host are influenced by dietary fiber intake as well as consumption of SCFA-enriched foods (Yamamura et al., 2020). Depletions of certain SCFAs can have detrimental effects on the host. Below we summarize the key aspects of SCFAs’ involvement in host health.

## SCFAs Regulation of Intestinal Immune Regulation

**Figure 1:**
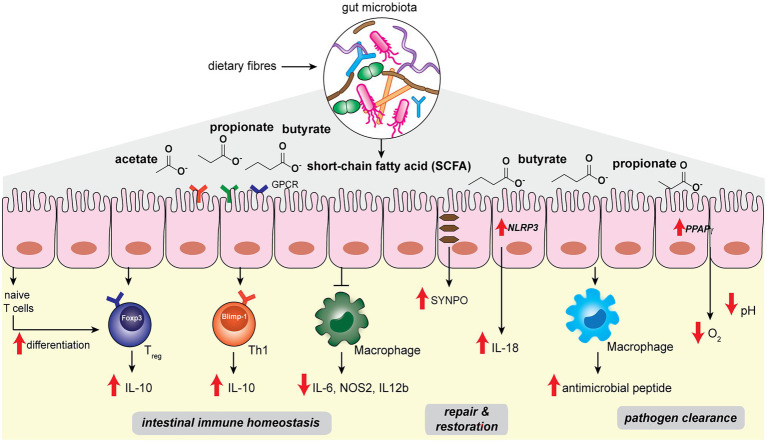
Gut microbiota-derived short-chain fatty acids such as acetate, propionate, butyrate regulate host intestinal immune homeostasis, epithelial repair and restoration, and facilitate pathogen clearance in the host gut niche.

The host intestinal immune system has co-evolved with the gut microbiota in order to maintain a delicate balance between tolerance to commensal microbes and defense against pathogenic ones (Hooper and Macpherson, 2010). Perturbation of the gut homeostasis can lead to intestinal inflammation and diseases such as inflammatory bowel diseases (IBD; Garrett et al., 2010; Maloy and Powrie, 2011). In the gut, the intestinal epithelial layer serves as the barrier that separates the host from abundant microbes in the luminal contents, while immune cells such as macrophages and T-cells residing in the lamina propria beneath the gut epithelial layer maintain active immunological surveillance (Figure 1; Varol et al., 2010). Abundant microbial SCFAs in the gut lumen modulates the colonic immune system to establish and maintain host intestinal homeostasis. Colonic regulatory T cells (cT_reg_) play the key role in modulating intestinal homeostasis and suppressing inflammation by reducing the proliferation of effector CD4^+^ T cells (T_effector_; Atarashi et al., 2011; Geuking et al., 2011). SCFAs such as propionic acid and butyric acids, when fed to specific pathogen-free (SPF) mice, can directly increase cT_reg_ *forkhead box P3 (Foxp3)* and interleukin-10 (*Il-*10) expression and enhance cT_reg_ differentiation, which offers beneficial adaptive immune responses to the host (Figure 1; Atarashi et al., 2013; Furusawa et al., 2013; Smith et al., 2013). Propionate is recognized by cT_reg_ *via* G-protein-coupled receptor 43 (GPCR43), encoded by the *Ffar2* gene, in a receptor-mediated process to inhibit histone deacetylase (HDAC) activities that control downstream gene expressions (Smith et al., 2013). On the other hand, butyrate is likely to stimulate other GPCRs on myeloid cells to promote T_reg_ cell differentiation (Furusawa et al., 2013). In addition, intronic enhancer conserved noncoding sequence 1 (CNS1)-dependent extrathymic differentiation of T_reg_ cells is also under the regulation of commensal bacteria-derived propionate and butyrate (Arpaia et al., 2013). Recently, SCFAs have also been shown to directly regulate T_effector_ by promoting microbiota antigen-specific T-helper 1 (Th1) cell Il-10 production (Sun et al., 2018). SCFAs interact with GPR43 and activate signal transducer and activator of transcription 3 (STAT3) and mammalian target of rapamycin (mTOR) pathways in Th1 cells, resulting in the upregulation of transcription factor B lymphocyte-induced maturation protein 1 (Blimp-1) to mediate Il-10 production (Figure 1; Sun et al., 2018). SCFAs-mediated T_reg_ and T_effector_ cells production of Il-10, the major immunosuppressive cytokines, plays an essential role in maintaining intestinal homeostasis.

Among the different SCFA molecules, butyrate selectively exhibits anti-inflammatory effects on bone marrow-derived macrophages (BMDM) and colonic lamina propria macrophages (Chang et al., 2014). Specifically, butyrate acts as an inhibitor of HDAC in BMDM to affect the expression levels of downstream proinflammatory genes such as *Il-6*, *Nos2*, and *Il-12b* (Figure 1). Although SCFAs are known to be transported into colonic epithelial cells *via* monocarboxylate transporters MCT1 and MCT4 (Stein et al., 2000; Wang et al., 2006), the uptake mechanism of butyrate into macrophages appears to be different (Chang et al., 2014). Future studies are needed to elucidate the exact pathways of butyrate transportation in macrophages. The ability of microbial butyrate to dampen the proinflammatory response in macrophages effectively prevents overstimulation of the intestinal immune system by commensal bacteria.

Another important factor for maintaining intestinal homeostasis lies in the ability of intestinal epithelial cells (IECs) to replenish and restore the barrier function in the face of inflammatory insult and damage (Peterson and Artis, 2014). Microbial SCFAs play a significant role in promoting IEC restitution. High-fiber diets and acetate treatment in SPF mice showed protective effects on gut epithelial integrity (Macia et al., 2015). Mechanistically, acetate binds to GPR43 and stimulates the K^+^ efflux in epithelial cells, leading to NLRP3 inflammasome activation and *Il-18* production, which are well-recognized pathways for promoting epithelial integrity, repair and homeostasis (Figure 1; Zaki et al., 2010). On the other hand, butyrate mediates epithelial integrity *via* different pathways. Wang et al. (2020) performed single-cell RNA sequencing in T84 cells treated with a physiological amount of butyrate. The actin-binding protein synaptopodin (SYNPO), a critical intestinal tight junction protein (Mun et al., 2014; Kannan and Tang, 2015), was identified as a top target whose expression is specifically induced by butyrate (Figure 1). The characterization of SYNPO establishes the mechanistic basis of a new aspect of butyrate’s role in maintaining intestinal homeostasis.

## SCFAs’ Role in Pathogen Clearance

Short-chain fatty acids offer host protection against enteric pathogens, as the depletion of SCFA-producing commensal bacteria by oral antibiotic treatment increases the likelihood of pathogenic growth in the host gut niche (Rivera-Chávez et al., 2016). On one hand, SCFA molecules can directly exert effects to limit the growth of pathogenic bacteria (Figure 1). For instance, by performing fecal microbiota transplantation from inbred mice that harbor diverse microbiota communities and manifest distinct infection kinetics, Jacobson et al. (2018) identified that abundant *Bacteroides* spp., such as *B. ovatus*, *B. uniformis* and *B. acidifaciens*, in commensal microbiota offers a high level of colonization resistance against pathogenic *S. Typhimurium*. Mechanistically, propionate produced by the *Bacteroides* spp. sufficiently acidifies the cytosolic environment of *S. Typhimurium* and reduces its lag phase growth, hence limiting the intestinal colonization of *S. Typhimurium* pathogens. The direct antibacterial effects of propionate are supported by the observations that (1) colonization of a *Bacteroides* mutant lacking propionate production ability did not offer protection, and (2) inulin-propionate ester treatment in mice, which specifically raises the level of propionate, augmented the colonization resistance (Jacobson et al., 2018). On the other hand, SCFAs can also indirectly enhance the host gut epithelial and immune cell functions for pathogen clearance. Butyrate produced by commensal microbes activates the host epithelial peroxisome proliferator-activated receptor-γ (PPAPγ) signaling pathway, promoting beta-oxidation of the colonic epithelial cells to reduce the oxygen availability in the gut lumen (Figure 1; Byndloss et al., 2017). Depleting butyrate-producing strains results in the increase in nitrate and oxygen levels in the gut, which acts in cooperation with the reduced cT_reg_ population and increased inflammation, favoring the growth of facultative anaerobic *Enterobacteriaceae* pathogens, including *Escherichia coli*, *S. enterica*, and *S. Typhimurium* (Byndloss et al., 2017). In addition, butyrate also promotes antimicrobial peptides production during macrophage differentiation. During the macrophage colony-stimulating factor (M-CSF)-triggered differentiation of peripheral blood-derived CD14^+^ monocytes, the presence of butyrate significantly increased the antimicrobial function of macrophages (Figure 1; Schulthess et al., 2019). Metabolomics analysis of butyrate-treated macrophages showed reduced glycolysis, higher adenosine monophosphate (AMP) levels and mTOR inhibition, which are opposite of the pro-inflammatory effects of lipopolysaccharides (LPS). Single-cell RNA sequencing analysis revealed five clusters of butyrate-induced gene expression differences, including lysosomal and antimicrobial signatures *via* inhibition of the HDAC3 pathway (Schulthess et al., 2019). The effects of butyrate produced by commensal bacteria on macrophages may be involved in preventing intestinal and systemic infections in the host.

## SCFAs Involvement in Gut-Brain Crosstalk

Short-chain fatty acids produced by the gut microbiota are involved in gut-brain communication *via* multiple pathways. The review by Dalile et al. (2019) provided a comprehensive picture of the role of SCFAs in modulating brain function *via* immune, endocrine, neural, and humoral routes. Recently, Kundu et al. (2019) showed that young germ-free mice manifest increased neurogenesis in the brain after being transplanted with the gut microbiota from aged mice that contained higher proportions of butyrate-producing microbes. Correspondingly, administration of sodium butyrate directly to young germ-free mice results in similar beneficial effects, possibly *via* the regulation of brain-derived neurotrophic factor (BDNF) expression in hippocampus (Varela et al., 2014; Barichello et al., 2015). In the α-synuclein (αSyn) overexpressing Parkinson’ disease (PD) mice model, however, SCFAs can sufficiently increase neuroinflammation and motor dysfunction (Sampson et al., 2016). Moreover, transplantation of fecal microbiota from Parkinson’s disease patients to the αSyn-expressing mice recapitulates the PD pathophysiology. Thus, identifying specific SCFAs in PD patients may offer key insights into disease aetiology and biomarker discovery.

## Bile Acids

Bile acids represent an important class of metabolites in the reciprocal communications between the microbiota and the host. Synthesized from cholesterol in the host liver, primary BAs are stored in the gallbladder and secreted into the duodenum, assisting with the absorption of lipids and fat-soluble vitamins into the host (Joyce and Gahan, 2016). While the majority of the BAs are reabsorbed in the small intestine and recirculated back to the liver, about 5–10% enter the host colon reaching a concentration of 200–1,000 μM (Nguyen et al., 2018), where they undergo quantitative biotransformation by the actions of the gut bacterial enzymes (Ridlon et al., 2006). These microbiota-modified BAs are known as secondary BAs. Apart from their roles in digestion, both primary and secondary BAs are important hormones that interact with host nuclear receptors, including farnesoid X receptor (FXR) and G protein-coupled receptors to influence multiple aspects of host metabolisms, cancer progression, and immunity (Jia et al., 2018; Lavelle and Sokol, 2020; Mohseni et al., 2020; Quinn et al., 2020). Below we focus on recent studies on the roles of microbial bile salt hydrolase (BSH) and functions of specific BA metabolites in host gut homeostasis.

## Gut Microbiota BSHs in Secondary BAs Diversification

**Figure 2:**
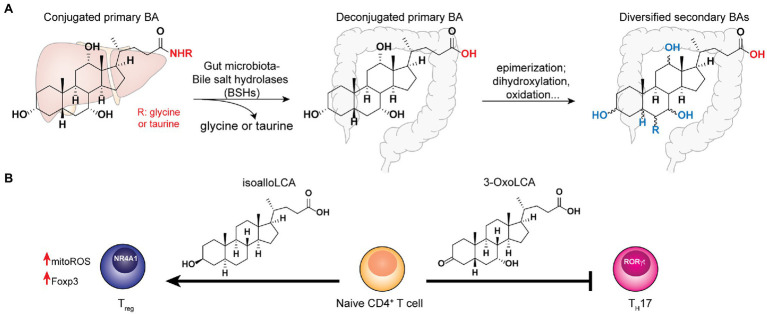
Bile acids synthesized in the host liver undergo bile salt hydrolases (BSHs) reaction followed by diverse biotransformation by the gut microbiota to become a variety of secondary bile acid metabolites that play key functions in host homeostasis. **(A)** The co-metabolism of bile acid in host and gut microbiota. **(B)** The distinct effects of specific secondary bile acids to T-cell differentiation.

The host-synthesized bile acids are hydroxylated C24 cyclopentane-phenanthrene sterols conjugated to either glycine or taurine (Russell, 2003). In the gut, the conjugated bile acids encounter the commensal microbes, many of which encode the bile salt hydrolases that hydrolyze the amide bond at the C24 position to yield unconjugated BAs (Ridlon et al., 2006). BSH hydrolysis is a key step that enables further microbial biotransformation including dihydroxylation, oxidation, epimerization of hydroxyl groups, and conjugation/deconjugation of amino acids to introduce structural diversities to secondary BAs (Figure 2A). The importance of microbial BSH activities in BA homeostasis and host metabolism is elegantly demonstrated in several recent studies. Mono-colonization of BSH-expressing *E. coli* strains into gnotobiotic mice resulted in a significant reduction of total plasma BAs and a specific reduction in tauro-conjugated BAs (Joyce et al., 2014). In addition, it also had a broader impact on host gene expression profiles and physiology, including reduction of weight gain, plasma cholesterol and liver triglycerides. Yao et al. (2018) further identified the BSH from commensal *Bacteroides thetaiotaomicron* has the selective activity of hydrolyzing tauro-*β*-muricholic acid. Remarkably, the expression of this single gene and enzymatic activity in the host can alter host metabolisms in both local and systemic levels in mouse models, demonstrating the functional importance of a major BSH from commensal *Bacteroides*. On the other hand, despite the wide conservation of BSHs in major gut bacterial genera *Bacteroidetes* and *Firmicutes*, the microbiota BSH enzymes showcase vastly different substrate specificity (Kawamoto et al., 1989; Yao et al., 2018). In addition to the BSH-expressing bacteria in the host, the bile acid 7-*α*-dehydroxylating gut bacteria, *C. scindens*, and *C. sordellii*, produce secondary BAs that potentiate the effects of endogenous antibiotics in killing pathogenic *C. difficile in vitro* (Kang et al., 2019). Taken together, understanding the specific functions of individual BSH and other BA-modification enzymes *in vivo* may identify potential avenues to fine-tune the host’s BA profiles to benefit the host.

## BAs Regulations of Host Innate and Adaptive Immunity

The diverse BAs function as either agnostic or antagonistic ligands of host receptors, influencing both the innate and adaptive immune pathways to modulate host homeostasis. It is well-established that BAs activate FXR receptors to regulate metabolisms. For example, deoxycholate is a specific BA found to promote colon crypt regeneration *via* activation of FXRs and their downstream innate immune pathways, which is temporally and reciprocally regulated with prostaglandin E2 (PGE2) during the colon wound healing process (Jain et al., 2018). Importantly, ulcerative colitis (UC) patients manifest a reduction of specific secondary BAs, lithocholic acid and deoxycholic acid (Sinha et al., 2020). Administrations of these metabolites at physiologically relevant concentrations afford anti-inflammatory roles in mouse colitis models, likely *via* immune cells’ TGR5 receptors (Sinha et al., 2020). In addition, BAs have also been shown to inhibit NLPR3 inflammasome *via* the TGR5-cAMP-PKA pathway to mitigate pathogen infections and inflammations in the host (Guo et al., 2016). Moreover, Ma et al. (2018) revealed that the pools of primary and secondary BAs in the host gut are carried by the portal vein into the liver, affecting hepatic natural killer T (NKT) cell accumulation and liver tumor growth, representing a new aspect of the crosstalk between gut microbiota-mediated BA metabolisms and host innate immunity.

In the context of adaptive immunity, BAs have been shown to regulate T cells functions. Song et al. (2020) established that the gut BA pools from dietary intake and microbial derivatizations affect a distinctive population of RORγ-expressing colonic FOXP3^+^ cT_reg_ cells, which play essential roles in maintaining colonic homeostasis and the amelioration of colitis severity in the host. Genetic deletions of BSH in the gut symbionts, *B. theaiotaomicron* and *Bacteroides fragilis*, reduce their abilities to trigger colonic RORγ+ T_reg_ cell differentiation specifically, without affecting other types of T_reg_ cells in mice. Interestingly, the dominant BA species likely exert their effects by interacting with the BA-sensing nuclear receptor, vitamin D receptor (VDR) that is abundantly expressed in colonic tissues (Song et al., 2020). To determine the specific roles of individual BAs in T cell regulation, Hang et al. (2019) screened a panel of 30 BAs for their effects on Th17- and T_reg_-cell differentiations from CD4^+^ T cells and established that 3-OxoLCA inhibits Th17 cell differentiation, while isoalloLCA promotes T_reg_ cell differentiation both *in vitro* and *in vivo* (Figure 2B). Mechanistically, 3-OxoLCA directly binds to the retinoid-related orphan receptor-γt (RORγt) transcription factor to inhibit Th17 differentiation, whereas isoalloLCA increases the production of mitochondrial reactive oxygen species (mitoROS) to trigger FOXP3 expression, hence enhancing T_reg_ cell differentiation. Notably, isoalloLCA promotes T_reg_ differentiation *via* the intronic Foxp3 enhancer, CNS3, which is distinct from the CNS1-dependent pathway mediated by SCFA butyrate (Arpaia et al., 2013). Using RNA-seq and ATAC-seq to interrogate the transcriptomic and genomic changes in isoalloLCA-treated naïve CD4^+^ T cells, Li et al. (2021) recently identified the NR4A family nuclear receptor, NR4A1, from the NR4A as a nuclear receptor, that is required for the immune-modulatory effects of isoalloLCA on T_reg_ differentiation. Understanding the effects of gut microbiota-derived BAs individually as well as synergistically in modulating host T cell functions are critical for biomarker discovery and therapeutics development for gastrointestinal inflammatory diseases.

## Peptidoglycan Fragments

**Figure 3:**
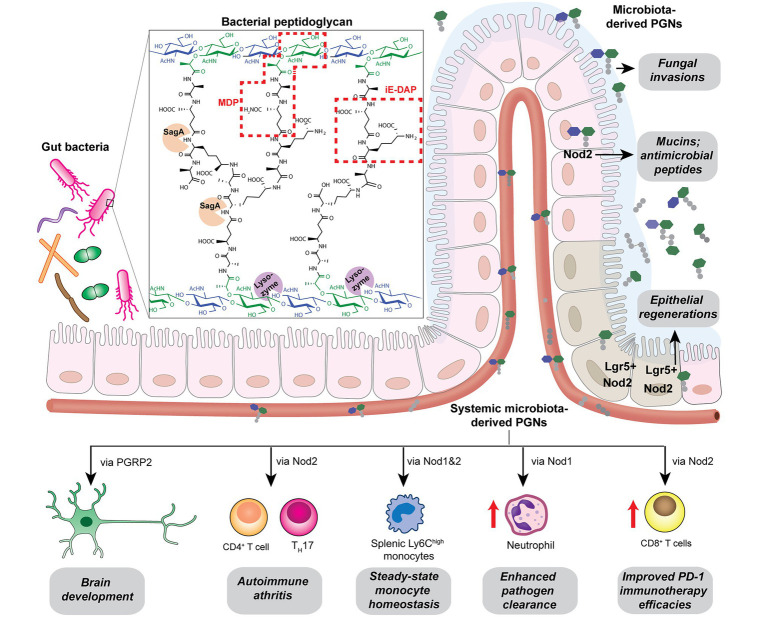
Gut microbiota-derived peptidoglycan fragments (PGNs) in the host gut niche as well as in systemic circulation act to modulate host homeostasis in multiple aspects.

Peptidoglycan is a major component of the bacterial cell wall that serves as a polymeric exoskeleton surrounding the cytoplasmic membrane in bacteria, preventing cell lysis due to the high turgor pressure (Vollmer et al., 2008). Unlike lipopolysaccharide (LPS, also known as endotoxin) which only exists in Gram-negative bacteria, peptidoglycan is an essential structure that is conserved across all bacteria, including Mycobacteria, Gram-positive bacteria and Gram-negative bacteria. The peptidoglycan polymer is made of long glycan strands composed of alternating *N*-acetyl glucosamine (GlcNAc) and *N*-acetyl muramic acid (MurNAc) *via* a *β*-1,4-linkage, with a short-stem peptide attached to each MurNAc residue. Adjacent stem peptides are further cross-linked to give strength to the peptidoglycan polymer. While the overall peptidoglycan composition is relatively conserved, the identity of the third amino acid of the stem peptide can differ significantly across bacteria (Table 1). Gram-positive bacteria typically possess an L-Lys at the third position of the stem peptide, which is further attached to a specific branch peptide; whereas most Gram-negative bacteria have a unique meso-diaminopimelic acid (mDAP) as the third amino acid in the stem peptide. In addition, some bacteria modify their peptidoglycan glycan backbone, such as 6-*O*-acetylation of MurNAc, N-deacetylation of GlcNAc and/or MurNAc. Albeit structurally complex, peptidoglycan is not static but undergoes constant remodeling and turnover processes that accommodate cell growth and division (Do et al., 2020; Egan et al., 2020). As a result, bacteria shed soluble peptidoglycan fragments (also known as muropeptides) into the environment during normal growth (Figure 3). Due to their unique presence in bacteria, PGNs are recognized by the mammalian nucleotide-binding oligomerization domain 1 and 2 (NOD1 and NOD2) as pathogen-associated molecular patterns (PAMPs) that activate the host’s innate immune defense in times of bacterial infections (Wolf and Underhill, 2018). The minimal motifs of peptidoglycan that activate NOD1 and NOD2 have been elucidated: the dipeptide, *D-γ*-Glu-mDAP (iE-DAP) is required for NOD1 activation, and the muramyl dipeptide, MurNAc-L-Ala-D-Glu/Gln (MDP) activates NOD2 (Girardin et al., 2003a,b). However, despite the wide applications of these synthetic ligands, whether the natural PGNs in host exist in the forms of iE-DAP or MDP have not been elucidated. The trillions of commensal bacteria that coexist with the human host likely generate a multitude of PGNs, which have been shown to circulate in human bloodstream ubiquitously (Huang et al., 2019). Recent studies have shed light on the emerging roles of the gut microbiota-derived PGNs as the newest family of microbiota metabolites in maintaining multiple aspects of host health and physiology beyond infections.

**Table 1:** Summary of bacterial peptidoglycan compositions.

Conserved PG structure	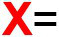	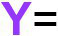	Bacteria*
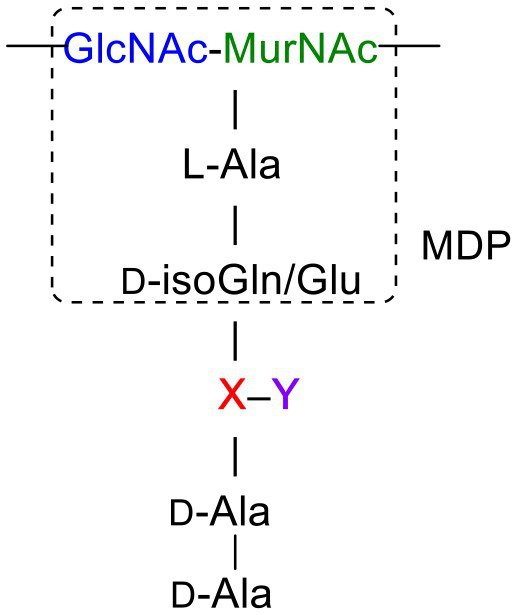 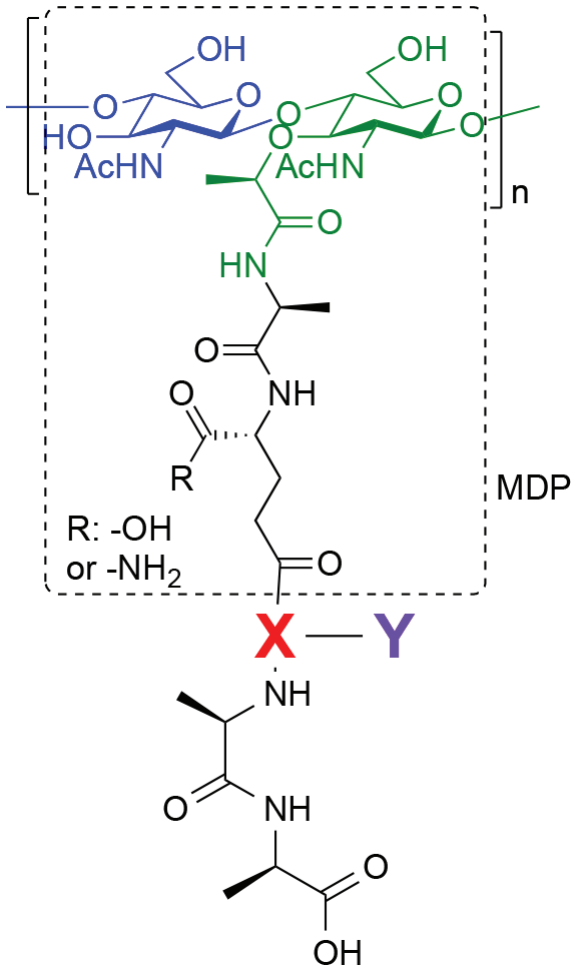	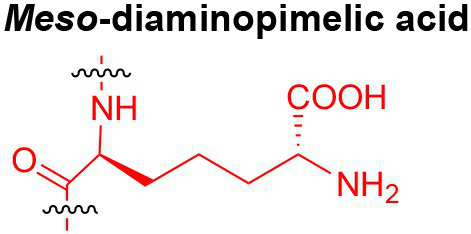	Nil	*Most Gram-negative bacteria*, e. g. *Escherichia coli* (−), *P. aeruginosa* (−)
	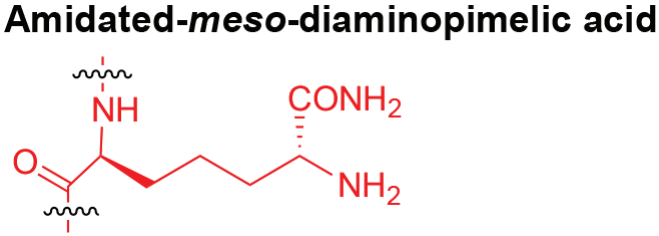	Nil	*B. subtilis* (+), *L. plantarum* (+), *B. anthracis* (+)
	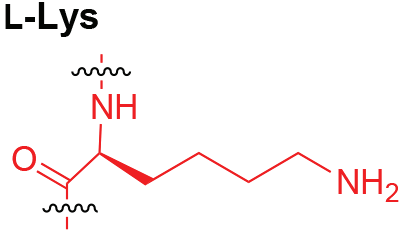	Nil	*Most Gram-positive bacteria, e.g. B. globosum* (+), *L. acidophilus* (+)
	Same as above	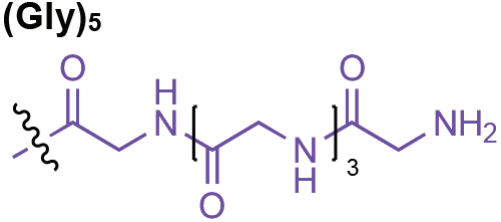	*S. aureus* (+)
	Same as above	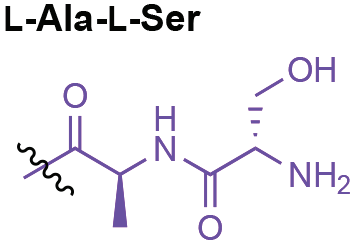	*Streptococcus pneumoniae* (+)
	Same as above	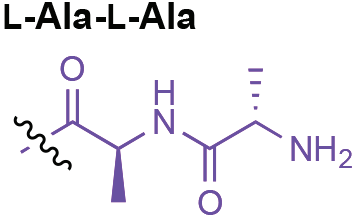	*E. faecalis* (+)	
	Same as above	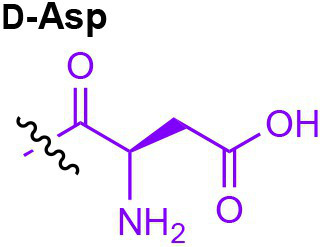	*Enterococcus faecium* (+), *L. lactis* (+), *L. casei* (+), *L. rhamnosus* (+)	
	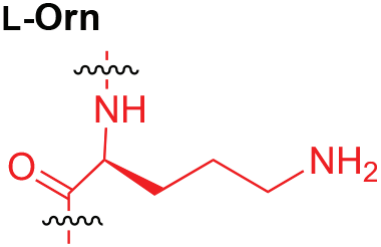	Nil	*B. globosum* (+)	
	Same as above	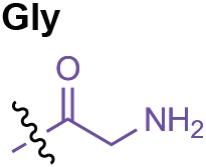	*B. burgdorferi* (+), *T. thermophilus* (–)	
	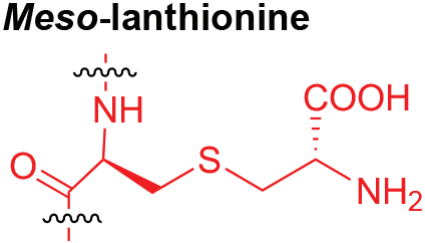	Nil	*F. nucleatum* (–)	
	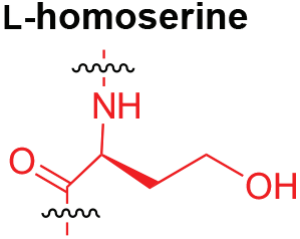	Nil	*C. poinsettiae* (+)	
	Same as above	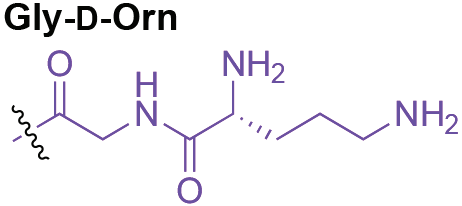	*Aureobacterium* spp. (+)	
	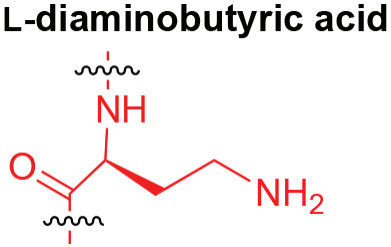	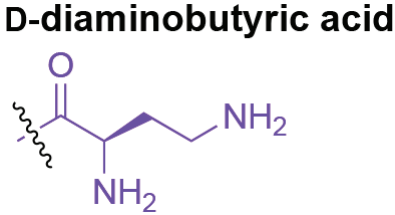	*C. aquaticum* (+)

*, +/− *indicates Gram-positive or Gram-negative bacteria*.

## Natural Adjuvants That Calibrate Host Immunity

Bacterial PGNs have long been recognized as vaccine adjuvants to boost host immune responses (Ellouz et al., 1974; Guryanova and Khaitov, 2021). The presence of gut microbiota-derived PGN molecules in healthy individuals raises the exciting prospects of their roles as endogenous adjuvants that calibrate host immunity. However, the existence of bacterial PGN in the human body had remained debatable in the era prior to microbiome research (Sen and Karnovsky, 1984). It was not until recently that Huang et al. (2019) developed a monoclonal antibody 2E7 that specifically recognizes muramyl-dipeptide (MDP), the minimal conserved motif of bacterial peptidoglycan, to enable sensitive ELISA detection of PGNs in host sera. Notably, PGN is undetectable in the serum of germ-free mice, supporting the gut microbiota being the origin of the systemic PGN in the host. The study revealed the ubiquitous presence of microbiota-derived PGNs in all human individuals, while the sera PGN levels significantly increased in patients of the autoimmune diseases rheumatoid arthritis and lupus. Huang et al. (2019) further demonstrated that 2E7-neutralization of the circulatory PGN alleviated arthritis progression in mouse models of the disease, highlighting the potential role of gut microbiota-derived PGNs in autoimmune disease progression. Thus, modulating the levels of microbiota-derived PGNs in the host offers a new prospect for the potential treatment of autoimmune diseases.

Another intriguing study by Griffin et al. (2021) unraveled that small muropeptides, produced by enterococcus peptidoglycan remodeling machinery, could act as natural adjuvants in the host to boost cancer immunotherapy. Commensal bacteria *Enterococcus faecium* and a few related species encode a secretory protein, antigen-A (Sag A), whose NlpC/p60 domain acts as an endopeptidase that truncates the stem peptide of peptidoglycan, releasing small muropeptides such as MDP and GMDP into the microbiota niche (Figure 3; Rangan et al., 2016; Kim et al., 2019). Administration of these immune-stimulating muropeptides remarkably improved the efficacy of anti-PD-L1 in treating B16-F10 melanoma in the mouse model (Griffin et al., 2021). Probiotic delivery of these immune-active PGN molecules into the host could provide an attractive avenue for tuning these natural immune adjuvants to improve therapeutic efficacies. It will be interesting to investigate if these microbiota-derived PGNs in the host are reliable biomarkers for predicting patients’ clinical response to anti-PD-L1 therapy.

## PGNs Regulation of Host Homeostasis

Like other microbiota-derived metabolites, PGNs produced by commensal bacteria play a vital role in maintaining intestinal homeostasis, exerting their impact either through direct effects on gut epithelium or indirect contributions to the host clearance of pathogenic microbes in the gut niche. Using MDP as a model PGN ligand, Nigro et al. (2014) demonstrated that microbiota-derived PGNs promoted the regenerations of the Lgr5^+^ stem cells in the mouse intestinal crypt *via* the NOD2-dependent pathway, which offers protective functions to the gut niche, especially under oxidative stress (Figure 3). This study establishes the beneficial role of microbiota-derived PGNs in the direct crosstalk in the intestinal crypt-microbiota interface. In addition, commensal bacteria engage specific PGN molecules to protect the gut niche by enhancing enteric tolerance of pathogenic bacteria. The abovementioned peptidoglycan hydrolase, SagA, produced by the commensal *E. faecium*, stimulates the mucin and antimicrobial peptide production in gut epithelium *via* MyD88 and NOD2-mediated innate immune pathways, rendering host gut resistance against *S. Typhimurium* and *Clostridium difficile* pathogenesis in the mouse model (Figure 3; Pedicord et al., 2016). The SagA-mediated protective effects on gut homeostasis are likely produced by specific bioactive muropeptides from gut bacteria by the NlpC/p60 hydrolase activity.

Apart from NOD2-dependent protective pathways in the gut, microbiota-derived PGN molecules also prime NOD1 signaling to enhance systemic defenses against pathogen infections. Clarke et al. fed mice with *E. coli* containing [^3^H]-DAP-labeled PGNs and detected radioactivity in the mouse serum and bone marrow cells, providing direct evidence for the translocation of DAP-containing PGN fragments from the gut microbiota into the systemic circulation (Weiser et al., 2010). The systemic microbiota-derived PGN molecules significantly facilitated neutrophil clearance of *Streptococcus pneumoniae via* a NOD1-dependent pathway, as no protective effect was observed in Nod1^−/−^ mice (Figure 3; Weiser et al., 2010). Consistently, administration of NOD1 ligands into germ-free mice can restore the hematopoietic stem cells in the bone marrow and stimulate cytokine productions, supporting that NOD1 signaling participates in the maintenance of steady-state hematopoiesis in the host (Iwamura et al., 2017).

Given that the levels and specific structures of PGNs in the host are important in maintaining host homeostasis, a perturbed level of the microbiota-derived PGNs may have detrimental effects. Oral administration of broad-spectrum *β*-lactam antibiotics, which inhibit bacterial peptidoglycan biosynthesis, has been shown to induce a sudden significant increase in PGN levels, known as “peptidoglycan storm,” in the gut of mice (Tan et al., 2021). The *β*-lactam-induced PGN storm profoundly impacts the bacteria-fungal interaction, strongly promoting the invasive hyphal growth and systemic dissemination of pathogenic *Candida albicans* in mice (Figure 3), hence establishing the underlying mechanistic basis for *β*-lactam antibiotics-associated fungal infections in the clinic.

## PGNs’ Involvement in Metabolic Diseases

**Figure 4:**
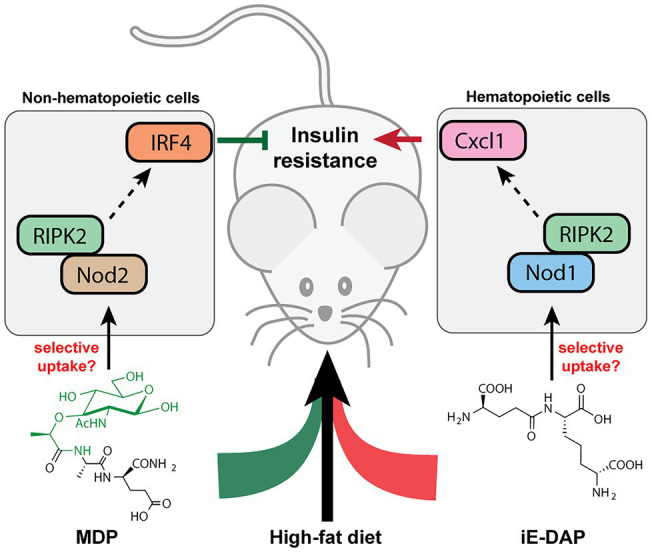
Peptidoglycan fragment agonists of NOD1 and NOD2 pathways manifest opposing effects on insulin resistance in high-fat diet mice. While MDP, a NOD2 ligand that triggers overexpression of IRF4 *via* PIPK2 in non-hematopoietic cells, ameliorates insulin resistance induced by high-fat diet in mice, the iE-DAP agonist of NOD1 pathways induces Cxcl1 expression *via* RIPK2 in hematopoietic cells that contributes to insulin resistance in high-fat diet mice.

Many metabolic diseases, including diabetes, manifest systemic low-grade chronic inflammation characterized by infiltrating immune cells such as macrophages and neutrophils into the metabolic tissues such as the liver, adipose tissues, and skeletal muscles (Fink et al., 2014; Morinaga et al., 2015). In particular, activation of the NOD innate immune pathways is pertinent in the immuno-metabolic interface and insulin resistance. In mice fed with a high-fat diet (HFD), their sera contain higher levels of NOD1-activating ligands, which are likely mDAP-containing PGNs that originated from the gut bacteria or diet (Chan et al., 2017). Administration of NOD1 ligands into mice triggers insulin intolerance without affecting the body weight or adiposity. Specifically, the NOD1 ligand-induced insulin resistance do not involve significant elevation of serum cytokines, which is in contrast to the response of low-dose LPS, the major contributor of metabolic endotoxemia (Schertzer et al., 2011). Hematopoietic deletion of NOD1 offered protective effects against diet-induced glucose and insulin intolerance in mice, which could be partially due to the decreased CXCL1 production by macrophages in the adipose tissue and hence reduced neutrophil migration (Figure 4; Chan et al., 2017). Understanding of neutrophil functions in NOD1-dependent whole-body insulin resistance and metabolic inflammations await further investigations. Consistently, the proinflammatory effects of NOD1 activators have been established in *in vitro* cell culture assays, where they trigger MAPK- and NF-kB-mediated proinflammatory gene expression such as *MCP-1*, *TNFα*, *Il-6* in adipose tissues (Zhao et al., 2011). Furthermore, Zhang et al. (2019) recently demonstrated that gut microbiota-derived NOD1-activating PGNs in the host circulatory system could directly modulate insulin trafficking in pancreatic beta cells. Mechanistically, PGN ligand binding to NOD1 in pancreatic beta cells triggers NOD1 and receptor interacting protein-2 (Rip2, also known as RIPK2) localization to insulin vesicles and recruits Ras-related protein Rab-1a for insulin transportation. This provides opportunities to further decipher the identities of these endogenous microbiota-derived NOD1 activators that regulate host glucose tolerance in the gut-islet axis.

Different from the widespread expression of NOD1 in most cell types, NOD2 is exclusively expressed in a limited number of cells such as intestinal epithelium and immune cells in mammals (Caruso et al., 2014). In parallel with the NOD1-mediated adipocyte and hepatocyte inflammations in insulin resistance, NOD2 activation by the corresponding PGN ligands in skeletal muscle cells also provoked insulin resistance *via* cell-autonomous innate immune responses (Tamrakar et al., 2010). Administration of MDP to myotube cells *in vitro* significantly impairs insulin-stimulated glucose uptake and activates proinflammatory cytokine release, suggesting a direct causal relationship between NOD2 activation and insulin resistance in skeletal muscle cells *in vitro*. However, whether such cell-autonomous proinflammation holds true in mice fed with NOD2 agonists remain to be investigated, since the interpretation of NOD signaling being universally proinflammatory may be an over-simplification of the *in vivo* effects (Cavallari et al., 2020). Cavallari et al. (2017) recently reported that the intraperitoneal administration of MDP into two different HFD-based obesity mice models surprisingly improved insulin and glucose tolerance without altering other parameters such as body mass, adiposity, serum endotoxin levels as well as gut microbiome composition. Mechanistically, MDP exerts its insulin-sensitizing effects in adipose and hepatic tissues *via* the activation of NOD2 and hence increased expression of interferon regulatory factor 4 (IRF4), a downstream transcription factor involved in anti-inflammatory response; whereas IRF4 was dispensable for the NOD1-mediated insulin resistance *via* hematopoietic cells (Cavallari et al., 2017). Interestingly, the common adapter protein Rip2 in both NOD1/2 signaling is involved in the dichotomous metabolic effects in different cell types (Figure 4; Cavallari et al., 2020). As a result, the cell-specific and ligand-specific NOD innate immune responses are complex and require multiple levels of regulations in conferring whole-body metabolic effects (Schertzer and Klip, 2011). For instance, the localization and compartmentalization of specific PGN agonists in various cells and tissues may be important for eliciting precise immune and metabolic responses (Cavallari et al., 2020). The selective uptake of NOD1 or NOD2 agonists by different cells may play a role in the divergent responses. Yet, the host’s transport mechanisms of microbiota-derived PGN molecules are beyond current knowledge (see next section). Moving forward, for developing PGN postbiotic therapeutics, a key challenge is to define how different immune-metabolic pathways respond to diverse arrays of microbiota-metabolites are orchestrated in the host. Addressing the compositions, distributions, and levels of natural gut microbiota-derived PGNs in individuals may offer new perspectives.

## PGNs in the Gut-Brain Axis

The presence of commensal microbiota-derived PGN molecules in the host circulatory system (i.e., serum and cerebrospinal fluid) under basal conditions establishes the necessary prerequisite for PGNs to reach and regulate extraintestinal organs such as the brain. Recent studies have shed insights on their involvement in normal brain development and chronic brain inflammations in diseases such as multiple sclerosis (MS) *via* the regulation of innate immune pathways in the brain (Gonzalez-Santana and Diaz Heijtz, 2020; Laman et al., 2020). Using an enzymatic colorimetric-based silkworm larvae plasma (SLP) assay to detect bacterial PGN, Arentsen et al. (2017) demonstrated that higher PGNs are present in the cerebellum of juvenile SPF mice than germ-free (GF) mice, validating that microbiota-derived PGNs can indeed cross the blood-brain barrier (BBB). Interestingly, the cerebellum PGN levels increased in an age-dependent manner, which parallels the gut microbiota colonization process in mice. Corroboratively, the expression of peptidoglycan-recognition protein 2 (PGRP2) and the putative PGN transporter PepT1 also significantly increases during this specific postnatal period in the developing brain and are sensitive to changes in the gut microbiota. PGRP2-mediated signaling is critical for brain development and behavior in mice, as PGRP2^−/−^ mice manifest altered expressions in synapse-related genes including autism risk gene *c-Met* and abnormal social behavior (Arentsen et al., 2017). This seminal study demonstrated the emerging role of microbiota-derived PGNs in the gut-brain axis. However, the lack of knowledge in the gut microbiota composition and PGN profiles in host impedes the establishment of direct causal effects. Further studies are needed for elucidation of the specific subtypes of PGN molecules recognized by PGRPs and to address their exact functions in the developing brain.

In chronic brain inflammatory diseases such as multiple sclerosis (MS) with its experimental autoimmune encephalomyelitis (EAE) model, the host blood-brain barrier is severely damaged, indicative of inflammatory activity, which, in principle, facilitates the translocation of systemic PGNs into the brain. Consistently, PGNs were found to be concentrated in lesion regions in postmortem MS patients as well as in live patients (Schrijver et al., 2001; Branton et al., 2016). Purified PGNs can promote EAE development in mice *via* NOD signaling to engage RIP2 and RICK in infiltrating dendritic cells (DCs), and neutralizing antibodies against circulating PGNs can ameliorate disease progression (Shaw et al., 2011). Notably, pathogenic bacterial infections are also sources of elevated systemic PGNs contributing to host inflammation. In the maternal-fetal interface, bacterial PGNs (and other bacterial cell wall products) from the mother can translocate across the placenta to trigger neural proliferation in the fetal brain and result in abnormal postnatal cognitive behaviors (Humann et al., 2016). These studies suggest the potential pathological functions of commensal and pathogenic bacteria-derived PGNs in brain inflammations. Future prospective studies to profile the gut microbiota composition and the microbiota-derived-PGNs in these patients may uncover valuable PGN biomarkers for brain disease diagnosis.

## Host Transporters for PGNs Uptake

As the mammalian innate immune NOD1 and NOD2 sensors reside in the cytosolic space in host cells, the soluble PGNs produced by the gut microbiota must find their ways into the cells for binding to the cognate NOD targets. Bastos et al. (2021) recently published a comprehensive review on the uptake and recognition of PGNs by mammalian hosts, below we highlight known mammalian transporters that may implicate in the transportation of natural microbiota-derived PGNs into host cells. The seminal work by Vavricka et al. (2004) established that the di/tripeptide hPepT1 of the solute carrier 15A1 (SLC15A1) family transports MDP into human colonic epithelial Caco2-BBE cells. Critically, the authors used radiolabeled [^3^H]-MDP to directly demonstrate the time- and concentration-dependent uptake in hPepT1-expressing cell lines. While hPepT1 is only minimally expressed in the colonic epithelial cells under normal physiological conditions, it is strongly overexpressed in chronic colonic inflammations such as IBD (Vavricka et al., 2004). Interestingly, its expression window in the brain tissues also coincides with the period of gut microbiota colonization in mice (Arentsen et al., 2017). On the other hand, whether hPepT1 also transports other PGNs still remains inconclusive: in the *in vitro* competition assay using hPepT1-expressing *Xenopus laevis* oocytes, GMDP and other NOD1-activating PGNs, unlike MDP, failed to inhibit the cellular uptake of a specific hPepT1 substrate, [^3^H]-Gly-Sar (Ismair et al., 2006), whereas opposite results were observed in a similar competition assay in Caco-BBE cells and lung epithelial cells (Dalmasso et al., 2010; Hu et al., 2018). As a result of the great challenges with obtaining substantial quantities of PGNs for biological assays, direct measurements using radiolabeled PGNs to validate the involvement of hPepT1 in their transportations are not feasible yet (Ismair et al., 2006). Additionally, a distantly related SLC46 family transporter in Drosophila has been suggested to transport tracheal cytotoxin (TCT), a natural PGN released by pathogenic *Bordetella pertussis* (Paik et al., 2017). Yet whether mammalian SLC46 also participates in PGNs uptake process has not been established in direct assays. Furthermore, fluorescent MDPs are valuable for visualization of the uptake process in host cells. For instance, specific endo-lysosomal transporters, SLC15A3 and SLC15A4, are found to mediate the selective endosomal escape of MDP, facilitating the recruitment of NOD2 sensor and downstream effectors onto endosomal platforms in mammalian dendritic and macrophage cells (Nakamura et al., 2014; Hu et al., 2018). Overall, while current studies indicate that the SLC15 family of transporters play prominent roles in canonical PGN ligands uptake, our understanding of the uptake process is far from clear. It is plausible that PGNs uptake occurs in a subtype-specific and cell-specific and manner, where distinct host cell types engage different mechanisms for diverse PGN subtypes. Hence, efforts to unravel the natural structures of microbiota-derived PGNs in host and to develop facile access to obtain them (and their probes) are urgently needed to facilitate the mechanistic elucidations.

## Other Microbiota-Derived Metabolites

### Tryptophan and Indole-Derivatives

**Figure 5:**
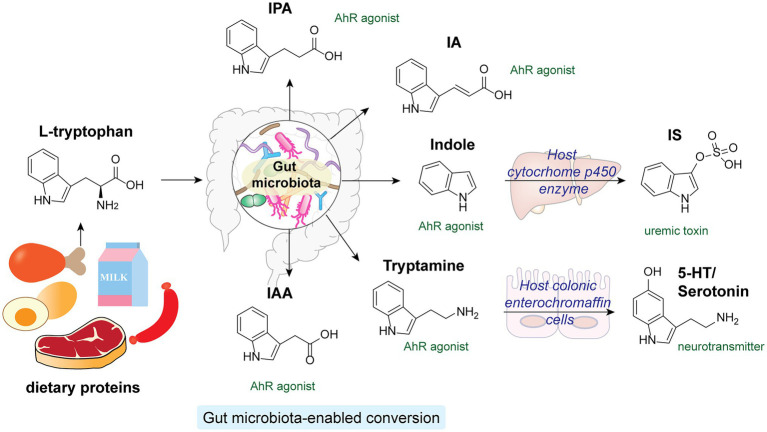
l-tryptophan from dietary proteins is metabolized by gut microbiota into a variety of indole derivatives, which are potent AhR agonists in the host.

Tryptophan is an essential amino acid that structurally contains an indole aromatic ring. As human enzymes do not synthesize tryptophan, it can only be obtained from dietary proteins (Roager and Licht, 2018). While the majority of the ingested proteins are absorbed in the small intestine in the host, a considerable amount of tryptophan and tryptophan-containing peptides can reach the colon, where the gut microbiota transform them into a diverse family of indole-derivatives, such as indole, indole-acetic acid (IAA) and indole-propionic acid (IPA; Figure 5; Geypens et al., 1997). The absence of indole-derivatives in germ-free mice indicates the involvement of microbiota in their production (Wikoff et al., 2009). Extensive studies have revealed key functions of microbiota-derived indole derivatives as signaling molecules in host metabolisms (Agus et al., 2018, 2021; Roager and Licht, 2018). In this section, we mainly focus on indole derivatives’ activation of the host’s aryl hydrocarbon receptor (AhR) pathway.

Aryl hydrocarbon receptor is a cytosolic transcription factor widely expressed in host immune cells and involved in various innate and adaptive immune responses upon activation by specific endogenous ligands and xenobiotics (Rothhammer and Quintana, 2019). Microbial-derived tryptophan and indole derivatives are agonists for AhR, yet their affinities toward AhR from mice and humans differ substantially (Hubbard et al., 2015). Interestingly, indole metabolites are significantly reduced in fecal samples from individuals with metabolic syndrome and HFD-fed mice. Supplementation of *Lactobacillus reuteri*, a high producer of indole derivatives, to HFD-fed mice was sufficient to restore AhR-dependent metabolic homeostasis, including insulin sensitivity, intestinal barrier function, and glucagon-like peptide-1 (GLP-1) hormone production (Natividad et al., 2018). In addition, specific indole-derivatives are shown to exhibit anti-inflammatory activities *via* AhR signaling (Figure 5). For instance, IAA can prevent HFD-induced antibiotics tolerance in mice by acting synergistically with bactericidal antibiotics such as ciprofloxacin, possibly by promoting bacterial metabolism and ROS production (Liu et al., 2021). IPA and indoleacrylic acid (IA) produced by *Peptostreptococcus russellii*, a mucin-utilizing bacteria, promote goblet cell functions *via* AhR signaling, hence boosting gut barrier functions and ameliorating inflammatory responses (Wlodarska et al., 2017). Furthermore, the indigenous spore-forming bacteria in the gut microbiota mediate the production of 5-hydroxytryptamine (5-HT), also known as serotonin, by promoting Thp1 tryptophan digestion in host colonic enterochromaffin cells (Yano et al., 2015). Apart from being an important neurotransmitter, serotonin hold key regulatory functions in governing host gut homeostasis. Local accumulation of serotonin in the gut contributes to its systemic translocation and uptake by circulating platelets, promoting host GI motility and global homeostasis (Yano et al., 2015). While most microbiota-derived indole derivatives offer beneficial effects, one notable exception is indoxyl sulfate (IS), which is converted from indoles by cytochrome P450 enzymes in the host liver (Brydges et al., 2021). A uremic toxin, IS accumulates significantly in the sera of chronic kidney disease patients. Excessive IS that activates AhR signaling in endothelial cells impairs angiogenesis, substantially increasing the chance of cardiovascular diseases (Dou et al., 2007; Schroeder et al., 2010; Wu et al., 2011).

Therefore, proper gut microbiota-host crosstalk that regulates both the concentration and diversity of the indole metabolites pool in the host is essential for host health and functioning. Although a wide range of tryptophan and indole ligands can activate AhR signaling, individual metabolites may elicit distinct downstream effects. Elucidation of the biological impact of specific microbiota-derived indole derivatives is necessary for developing therapeutic strategies targeting this pathway.

### Trimethylamine N-oxide

**Figure 6:**
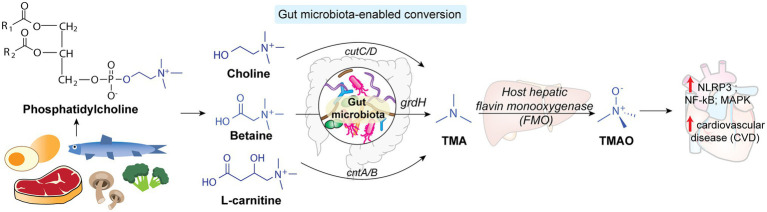
The gut microbiota is involved in converting dietary phosphatidylcholine into TMA, which is transported to the host liver and converted to TMAO that is strongly associated with cardiovascular disease in the host.

Trimethylamine N-oxide (TMAO) is a gut microbiota-derived metabolite first identified in human sera as a risk predictor for cardiovascular diseases in an untargeted metabolomics study in 2011 (Wang et al., 2011). The host gut microbiota plays the essential role in metabolizing dietary phosphatidylcholine, betaine, and l-carnitine precursors into trimethylamine (TMA). As illustrated in Figure 6, key microbial enzymes involved in TMA production include TMA lyase (CutC) and its activating protein (CutD; Craciun and Balskus, 2012), the seleonocysteine-containing betaine reductase (GrdH), and a two-component system composed of an oxygenase (CntA) and an associated reductase (CntB) (Zhu et al., 2014). Subsequently, TMA traverses across the intestinal epithelium into host systemic circulation and is carried to the liver, where the host hepatic flavin monooxygenase (FMO) converts TMA to TMAO (Yang et al., 2019). Circulatory levels of TMAO in individuals are closely associated with cardiovascular diseases (Witkowski et al., 2020). Mechanistically, TMAO promotes atherosclerosis and thrombosis by activating NLRP3 inflammasomes, NF-kB and MAPK signaling in vascular smooth muscle cells and endothelial cells, triggering proinflammatory gene expression and endothelial adhesions (Yang et al., 2019). Since the gut microbiota participates in a vital step in TMAO production in the host, inhibition of specific microbiota enzymes may effectively regulate TMAO levels in the host. Notably, the unique activity of microbial CutC/D enzymes that cleave the C-N bond of choline is absent in the mammalian counterpart, making them attractive targets for inhibition. The natural product, 3,3-dimethylbutanol (DMB) has been shown to be a potent inhibitor of CutC/D, which effectively reduces circulating TMAO and thwarts atherosclerosis in a mouse model (Wang et al., 2015). Certain halomethylcholines can irreversibly inhibit microbial TMA lysate gene *via* a suicide substrate mechanism (Roberts et al., 2018). Remarkably, a single dose of the inhibitor effectively reduced plasma TMAO in mice for a sustained period without exerting host toxicity (Organ et al., 2020). This study exemplifies the promise of modulating the microbiota-host crosstalk by selectively targeting microbiota enzymes to mitigate host diseases. In addition, dietary interventions with prebiotics, probiotics and other bioactive or phytochemical compounds represent another promising approach to modulate the production of gut microbiota-derived TMAO, hence suppressing the circulating levels of TMAO in host (Simó and García-Cañas, 2020).

### Polysaccharide A

Polysaccharide A (PSA), which belongs to one of the eight structurally distinct capsular polysaccharides (CPS) in the major commensal and non-enterotoxigenic bacteria *B. fragilis*, has been shown to exhibit considerable beneficial effects to human health (Ramakrishna et al., 2019; Vernay et al., 2020). PSA is the most well-recognized immunomodulatory zwitterionic carbohydrate molecule from the gut microbiota (Surana and Kasper, 2012). Mono-colonization of GF and SPF mice with either PSA-producing *B. fragilis* strains or purified PSA molecules affords an increase of CD4^+^ T lymphocyte populations and corrects Th1/Th2 imbalance in the mice spleen, supporting the role of microbiota zwitterionic PSA as a potent T cell-dependent antigen (Mazmanian et al., 2005). Mechanistically, *B. fragilis* PSA is recognized by CD11c^+^ DCs residing in the colonic lamina propria to reach the mesenteric lymph nodes (MLNs) and stimulate T cell responses. This occurs without *B. fragilis* PSA ever reaching the systemic circulation in the host (Mazmanian et al., 2005). Moreover, Round et al. (2011) discovered that colonization of GF mice with non-PSA-expressing *B. fragilis* resulted in a significant increase in the proinflammatory Th17 cell responses in the gut, which is absent in mice colonized with wild-type *B. fragilis*, suggesting that PSA performs an active symbiotic function in suppressing proinflammation and promoting immunologic tolerance in the host. Specifically, PSA signals Foxp3^+^ through TLR2 in T_reg_ cells to induce anti-inflammatory Il-10 cytokine production in the gut. It is surprising that, unlike other known PAMP ligands that trigger proinflammatory responses *via* TLR2 signaling, PSA has evolved to promote symbiotic tolerance *via* TLR2 activation of T_reg_ cells instead. Therefore, defining specific functions of the gut microbiota-derived molecules in the host is of great importance. Furthermore, *B. fragilis* PSA has been shown to offer protection in the mouse model of colitis *via* both innate and adaptive immune pathways by triggering the plasmacytoid DCs (PDCs) *via* TLR2 activation to mediate colonic T_reg_ cells to produce anti-inflammatory IL-10 to dampen intestinal inflammation (Dasgupta et al., 2014). Besides the archetypal *B. fragilis* PSA of the gut microbiota, Neff et al. (2016) has performed a genome screen to uncover more immunomodulatory zwitterionic polysaccharides (ZPSs) encoded by other commensal bacteria. Such efforts to discover the structures and mechanisms of diverse microbiota-derived ZPSs will open up opportunities for potential therapeutic developments against intestinal inflammations of the host.

## Conclusion

Over the eons of evolution, the trillions of microbes residing in the human gut have developed a symbiotic relationship with the host, where the gut microbiota-derived metabolites serve as effector molecules modulating host functioning in the gut niche as well as in the extraintestinal organs. The gut microbiota possesses unique enzymatic capabilities to convert dietary molecules (i.e., dietary fibers and tryptophan-rich proteins) and host metabolites (such as conjugated primary bile acids), and *de novo* synthesize bacterial carbohydrate molecules (such as PGN fragments and PSA), producing a trove of microbiota-derived metabolites in the host. These microbiota-derived metabolites impact the host by acting as ligands for various receptors to stimulate downstream effects. In summary, characterization of the structures and functions of these microbiota-derived molecules in the host holds the promise to uncover the underlying mechanisms of gut microbiota-host crosstalk, opening up potential avenues for therapeutic interventions to microbiome-associated diseases in humans. We are in an exciting era that novel microbiota-derived metabolites are being uncovered from untargeted metabolomics and genome mining approaches. The challenge is to elucidate the biological roles of these chemical messengers and how they cause or contribute to various human diseases.

## Author Contributions

CL and YQ conceptualized the work. CL, YL, and YQ wrote the manuscript and made the figures. All authors contributed to the article and approved the submitted version.

## Funding

The work is supported by the Singapore National Research Foundation (NRF) under NRF fellowship award NRF-NRFF12-2020-0006, and a Nanyang Technological University start-up grant (NTU-SUG) to YQ.

## Conflict of Interest

The authors declare that the research was conducted in the absence of any commercial or financial relationships that could be construed as a potential conflict of interest.

## Publisher’s Note

All claims expressed in this article are solely those of the authors and do not necessarily represent those of their affiliated organizations, or those of the publisher, the editors and the reviewers. Any product that may be evaluated in this article, or claim that may be made by its manufacturer, is not guaranteed or endorsed by the publisher.
